# Macromolecular Crowding as a Tool to Screen Anti-fibrotic Drugs: The Scar-in-a-Jar System Revisited

**DOI:** 10.3389/fmed.2020.615774

**Published:** 2021-01-14

**Authors:** Nataly Puerta Cavanzo, Emilia Bigaeva, Miriam Boersema, Peter Olinga, Ruud A. Bank

**Affiliations:** ^1^Department of Pharmaceutical Technology and Biopharmacy, University of Groningen, Groningen, Netherlands; ^2^MATRIX Research Group, Department of Pathology and Medical Biology, University Medical Center Groningen, University of Groningen, Groningen, Netherlands

**Keywords:** macromolecular crowding, drug testing, fibrosis, collagen, myofibroblast

## Abstract

An unsolved therapeutic problem in fibrosis is the overproduction of collagen. In order to screen the effect of anti-fibrotic drugs on collagen deposition, the Scar-in-a-Jar approach has been introduced about a decade ago. With macromolecular crowding a rapid deposition of collagen is seen, resulting in a substantial decrease in culture time, but the system has never been tested in an adequate way. We therefore have compared six different macromolecular crowders [Ficoll PM 70 (Fc70), Ficoll PM 400 (Fc400), a mixture of Ficoll 70 and 400 (Fc70/400), polyvinylpyrrolidone 40 (PVP40), polyvinylpyrrolidone 360 (PVP360), neutral dextran 670 (ND670), dextran sulfate 500 (DxS500), and carrageenan (CR)] under profibrotic conditions (addition of TGFβ1) with primary human adult dermal fibroblasts in the presence of 0.5 and 10% FBS. We found that (1) collagen deposition and myofibroblast formation was superior with 0.5% FBS, (2) DxS500 and CR results in an aberrant collagen deposition pattern, (3) ND670 does not increase collagen deposition, and (4) CR, DxS500, and Fc40/700 affected important phenotypical properties of the cells when cultured under pro-fibrotic conditions, whereas PVP40 and PVP360 did less or not. Because of viscosity problems with PVP360, we conclude that PVP40 is the most optimal crowder for the screening of anti-fibrotic drugs. Finally, the effect of various concentrations of Imatinib, Galunisertib, Omipalisib or Nintedanib on collagen deposition and myofibroblast formation was tested with PVP40 as the crowder.

## Introduction

Most cells are embedded in a tissue-specific micro-environment composed of extracellular matrix (ECM) molecules. One of the structural components of the ECM is collagen. There are several types of collagen, with the fibrillar collagens (e.g., collagen type I, II, and III) being the most common ones. The precursor procollagen needs to be processed into mature collagen to enable the formation of supramolecular structures outside the cell. Especially in culture systems, this processing is time-consuming, and extended culture times are required to obtain a sufficient amount of extracellular matrix. Already in 1986 Bateman et al. (1) showed that the addition of neutral polymers in culture medium markedly enhanced the conversion of procollagen into collagen. They showed that the rate-limiting step in culture medium is the proteolytic cleavage of the N- and C-propeptides by propeptidases, and that adding polymers facilitated this proteolytic cleavage, which was confirmed by Hojima et al. (2). This system, which is due to macromolecular crowding (volume exclusion), has recently attracted considerable attention when it was re-invented in 2007 by Lareu et al. (3, 4).

Macromolecular crowding (MMC) is an easy and inexpensive tool to facilitate tissue engineering (5–7). It has been used to produce an extracellular micro-environment that boosts the potential of adult mesenchymal stem cells (8–13), such as enhancing adipogenic and osteogenic differentiation, and more effectively building constructs required for tissue engineering. Macromolecular crowding has further been used to improve (a) organotypic skin equivalents by promoting a functional dermal-epidermal junction in a condensed time frame (14), (b) the production of Bruch's membrane-like structures for culturing retinal pigment epithelial cells (15), and (c) bone and cartilage tissue engineering (16–18). Another interesting observation is, that the ECM produced by adult fibroblasts under MMC conditions is able to propagate human embryonic stem cells, even outperforming Matrigel (19). With respect to natural biomaterials, it was found that MMC not only enhances the polymerization rate of collagen type I, but also tunes fiber diameter and organization, a fact that can be explored for optimizing the properties of soft collagen hydrogels (20–23) or collagen films (planar constructs) (24, 25). Lastly, MMC has also been used for improving hydrogels derived from decellularized matrices (26).

A pathology with a serious burden to global health is fibrosis. At the molecular level fibrosis is, in essence, the accumulation of an excessive amount of ECM which mainly consists of collagen type I. It is stated that fibrotic phenomena play a major role in about 45% of all Western world death cases (27, 28). Despite decades of research, pharmacological treatments only modestly (if at all) attenuates the pathogenesis of fibrosis. Thus, there is a high need to develop anti-fibrotic drugs. Since the hallmark of fibrosis is the deposition of excessive amounts of collagen by activated fibroblasts (i.e., myofibroblasts) (29, 30), MMC might be a helpful tool for cellular drug screening, as it markedly shortens the culture time at which collagen deposition becomes visible. Indeed, MMC has been used for this purpose (5, 6, 31, 32), which is also known as the Scar-in-a-Jar system.

A variety of macromolecular crowders (MMCs) have been used to test the production of collagen by fibroblasts over time (3, 4, 6, 31–40). They are Ficoll PM 70 (Fc70), Ficoll PM 400 (Fc400), a mixture of Ficoll 70 and 400 (Fc70/400), polyvinylpyrrolidone 40 (PVP40), polyvinylpyrrolidone 360 (PVP360), neutral dextran 670 (ND670), dextran sulfate 10, dextran sulfate 500 (DxS500), carrageenan (CR), and polysodium-4-styrene sulfonate. Human fibroblasts used are WI-38 cells (embryonic lung fibroblasts) (3, 4, 6, 31, 38), WS-1 cells (embryonic dermal fibroblasts) (38, 39), adult dermal fibroblasts (37, 40), adult corneal fibroblasts (keratocytes) (34–36) and immortalized adult vocal fold fibroblasts (32). However, it should be stressed that it is the myofibroblast that is mainly responsible for the collagen deposition seen in fibrosis, not the fibroblast. As a consequence, testing of anti-fibrotic drugs with the Scar-in-a-Jar system should be done with myofibroblasts, or with fibroblasts that transform into myofibroblasts. The latter can be achieved by adding the strong profibrotic cytokine TGFβ1 into the culture medium (41, 42). The only reports that took this into consideration are those of Chen et al. (6, 31) and Graupp et al. (32), with DxS500 and Fc70/400 as MMCs. However, Chen et al. (6, 31) used embryonic lung fibroblasts; it is known that embryonic fibroblasts are phenotypically different from adult fibroblasts, as scarring does not occur in the embryo (43, 44). Also unfortunately, Graupp et al. (32) used immortalized adult vocal fibroblasts, not primary cells, and vocal fibroblasts are highly specialized cells. Thus, despite the advantages (short culture time in which collagen deposition as seen by optical analysis without the need for protein extraction) of the Scar-in-a-Jar principle, it seems that the system has never been tested or validated in a relevant setting.

We have compared the performance of six different MMCs (Fc70/400, PVP40, PVP360, ND670, DxS500, and CR) with primary human adult dermal fibroblasts under profibrotic conditions (presence of TGFβ1) in culture medium containing l-ascorbic acid 2-phosphate with 0.5 and 10% fetal bovine serum. These are the six MMCs that have been used in the past to accelerate collagen deposition (3, 4, 6, 31–40); the concentration used in our paper are the concentrations that have been recommended in the literature. We not only investigated collagen deposition, but also the morphology of the deposited collagen. We also tested the effect of the crowders on myofibroblast formation by means of alfa-smooth muscle actin staining. In addition, we investigated whether MMCs has an effect on the phenotypical properties of myofibroblasts. After all, if one wants to reliable test the effect of anti-fibrotic drugs, the phenotypical properties of the investigated cells should not be disturbed by the MMCs themselves. Finally, the performances of four anti-fibrotic drugs (Galunisertib, Omipalisib, Imatinib, and Nintedanib) were tested in the presence of PVP40.

## Materials and Methods

### Reagents and Antibodies for Cell Culture and Immunofluorescence

Reagents and final concentrations used in culture medium were as follows: Human recombinant TGFβ1 (5 ng/ml; 100-21C, Peprotech, London, UK); ascorbic acid (0.17 mM; A8960, Sigma-Aldrich); penicillin/streptomycin (pen/strep) (50 U/L; 15140122, Thermo Fisher Scientific, Landsmeer, the Netherlands). Macromolecular crowders were obtained from Sigma-Aldrich and used in the following concentrations in culture medium: Ficoll PM 70 (average molecular weight 70,000) (18.75 mg/ml; F2878; F2878; FVO 9%), Ficoll PM 400 (12.5 mg/ml; F4375; FVO 9%); Polyvinylpyrrolidone PVP-40 (average molecular weight 40,000) (21.5 mg/ml; PVP40; FVO 18%); Polyvinylpyrrolidone PVP-360 (average molecular weight 360,000) (11.34 mg/ml; PVP360; FVO 54%); Neutral Dextran 670 (analytical standard for GPC; average molecular weight 670,000) (100 μg/ml; 00896; FVO 5%); Dextran Sulfate 500 (sodium salt from *Leuconostoc* spp.; average molecular weight > 500,000) (100 μg/ml; D8906; FVO 5%); and Carrageenan CR (100 μg/ml; C1013; FVO 5%). The following drugs were used: Galunisertib (=LY2157299) (0.5, 1, 2.5 μM; 200-17, PeproTech); Omipalisib (=GSK2126458, GSK458, S2658) (0.01, 0.1, 1 μM; Selleck Chemicals); Imatinib (0.1, 0.5, 1 μM; I-5577, LC Laboratories, Woburn, MA); and Nintenadib (0.1, 0.5, 1 μM; Boehringer Ingelheim, Biberach, Germany). The used concentrations are in line with those of previous studies (45–49). Stock solutions of the drugs were prepared in dimethyl sulfoxide (DMSO; 100%) and stored at −20°C; the working solutions were diluted in culture medium with a final solvent concentration of ≤ 1%. The antibodies that were used are: mouse anti-human collagen type I (dilution 1:1,000; ab90395, Abcam, Cambridge, United Kingdom), mouse anti-human α-smooth muscle actin (α-SMA) (dilution 1:500; M0851, Dako, Glostrup, Denmark), and donkey anti-mouse IgG (H + L) Alexa Fluor 555 (dilution 1:1,000; A-31570, Thermo Fisher Scientific). The antibodies were diluted in PBS containing 2.2% bovine serum albumin (BSA) (K1106, Sanquin reagents, Amsterdam, the Netherlands).

### Cell Culture

Before the onset of experiments, normal adult primary human dermal fibroblasts (CC-2511, Lonza, Basel, Switzerland) were propagated in Dulbecco's modified Eagle medium (DMEM) (12-604F, Lonza) containing 50 U/L pen/strep and 10% fetal bovine serum (FBS) (Sigma-Aldrich). Cells were negative for mycoplasm contamination. At the start of the experiment, cells were trypsinized, reseeded at a density of 10.000 cells/cm^2^, and starved for 18 h in DMEM containing 50 U/L pen/strep, 0.5% FBS and 0.17 mM ascorbic acid. Experiments with the macromolecular crowders (Fc70/400, PVP40, PVP360, ND670, DxS500, or CR; see above for the used concentrations) were carried out in DMEM containing 50 U/L pen/strep, 0.17 mM ascorbic acid, 5 ng/ml TGFβ1 and 10 or 0.5% FBS. The prepared media was filtered before use through a SFCA 0.45 μm filter. For drug testing (Galunisertib, Omipalisib, Imatinib or Nintenadib; see above for the used concentrations), cells were cultured with the macromolecular crowder PVP40 dissolved in DMEM containing 50 U/L pen/strep, 0.17 mM ascorbic acid, 5 ng/ml TGFβ1 and 0.5% FBS in 24-well polystyrene plates (Costar) or 8-well Permanox chamber slides (ThermoFischer Scientific). Medium and compounds were refreshed every 24 h. It should be noted that cell density, MMC concentration and culture duration are comparable with the other studies in the field (3, 4, 6, 31–40).

### Immunofluorescence

Detection of deposited (=extracellular) collagen type I was carried out on cells that were washed twice with PBS and fixed with 4% paraformaldehyde (Sigma-Aldrich) for 10 min. Detection of both intracellular and extracellular collagen type I was carried out on fixed cells that were permeabilized with 0.5% Triton X-100 in PBS for 10 min. For immunofluorescence of α-smooth muscle actin, cells were washed twice with PBS and fixed with ice-cold methanol/acetone (1:1) for 10 min. Methanol/acetone fixed cells were first dried and later rehydrated with PBS before use. For all immunostainings, fixed cells were subsequently incubated with PBS containing 2.2% BSA for 30 min at RT. The primary mouse anti-human collagen type I and mouse anti-human α-SMA antibodies were incubated for 1 h at RT. The secondary antibody donkey anti-Mouse IgG (H + L) Alexa Fluor 555 was incubated for 1 h at RT. Nuclei were visualized with 4′,6-diamidine-2′-phenylindole dihydrochloride (DAPI) (1 μg/ml). All wash steps were performed in PBS. The cells were mounted with Citifluor AF1 (AF1-25, Brunschwig Chemie, Amsterdam, the Netherlands). Microphotographs were acquired in a random blind fashion with the use of a Leica DMRA microscope (Leica Microsystems, Rijswijk, the Netherlands) and a TissueFAXS microscopy system (TissueGnostics, Vienna, Austria).

### Immunofluorescence Quantification by ImageJ-Fiji

For collagen type I, 36 images (20x magnification) were obtained per well from a 24-well plate (total imaged area = 13.33 mm^2^). For α-SMA, 6 images (40x magnification) were acquired per well of an 8-well chamber slide (total imaged area = 1.72 mm^2^). Triangle automatic thresholding was applied to discriminate between fore- and background and the resulting mean fluorescence intensity of collagen and α-SMA present in each well was used for statistical analysis.

### RNA Extraction and Real-Time PCR

For gene expression analysis, total RNA was isolated with the Tissue Total RNA mini kit (Favorgen Biotech Corp., Taiwan). RNA quantity and quality were determined by UV spectrophotometry (NanoDrop Technologies, Wilmington, DE). One microgram of RNA was reverse transcribed with RevertAid First Strand cDNA Synthesis kit (Thermo Scientific). Real-time PCR was performed with SYBR green PCR master mix (Roche, Basel, Switzerland) and VIIA7 thermal cycling system (Applied Biosystems, Carlsbad, CA). Thermal cycling conditions were 2 min at 95°C (enzyme activation), followed by 15 s at 95°C, 30 s at 60°C and 30° s at 72°C (40 cycles). Melting curve analysis was performed in order to verify the absence of primer dimers. Primers were designed and optimized to have calculated 95–105% reaction efficiency; the used sequences are shown in Table 1. All data were normalized against the reference gene tyrosine 3-monooxygenase/tryptophan 5-monooxygenase activation protein, zeta isoform (*YWHAZ*) (50). The following genes were measured: the extracellular matrix proteins collagen type I, III, V and fibronectin (*COL1A1, COL3A1, COL5A*, and *FN1EDA*, respectively), the collagen chaperone heat-shock protein 47 (*SERPINH1*), the collagen modifying enzyme lysyl hydroxylase 2 (*PLOD2*), the collagen degradation enzymes matrix metalloproteinase 1 and cathepsin K (*MMP1* and *CTSK*), the cytoskeletal protein alfa-smooth muscle actin (*ACTA2*) and the endoplasmic reticulum stress response protein X-box binding protein 1 (*XBP1*).

**Table 1:** Primer sequences used for q-PCR.

**Gene**	**Forward sequence 5^**′**^3^**′**^**	**Reverse sequence 5^**′**^3^**′**^**
*COL1A1*	GCCTCAAGGTATTGCTGGAC	ACCTTGTTTGCCAGGTTCAC
*COL3A1*	AGGGTGCAATCGGCAGTCCA	CAATGGCAGCGGCTCCAACA
*COL5A1*	CCTGGATGAGGAGGTGTTTG	CGGTGGTCCGAGACAAAG
*FN1EDA*	AATCCAAGCGGAGAGAGTCA	GGAATCGACATCCACATCAG
*SERPINH1*	GCGGGCTAAGAGTAGAATCG	ATGGCCAGGAAGTGGTTTG
*PLOD2*	GGGAGTTCATTGCACCAGTT	GAGGACGAAGAGAACGC
*MMP1*	GCTAACCTTTGATGCTATAACTACGA	TTTGTGCGCATGTAGAATCTG
*CSTK*	GCCAGACAACAGATTTCCATC	CAGAGCAAAGCTCACCACAG
*ACTA2*	CTGTTCCAGCCATCCTTCAT	TCATGATGCTGTTGTAGGTGGT
*XBP1*	GTGAGCTGGAGCAACAAGT	AGGCCATGAGTTTTCTCTCG
*YWHAZ*	GATCCCCAATGCTTCACAAG	TGCTTGTTGTGACTGATCGAC

### Statistical Analysis

All data are represented as standard error of the mean (SEM) of at least three independent experiments and were analyzed with GraphPad Prism version 7.02 (GraphPad Software, La Jolla, CA) by unpaired *t* test with Welch's correction. Values of *p* <0.05 (95% confidence interval) were considered to be statistically significant: ns = *P* > 0.05; ^*^ = *P* ≤ 0.05; ^**^ = *P* ≤ 0.01; ^***^ = *P* ≤ 0.001; ^****^ = *P* ≤ 0.0001.

## Results

**Figure 1:**
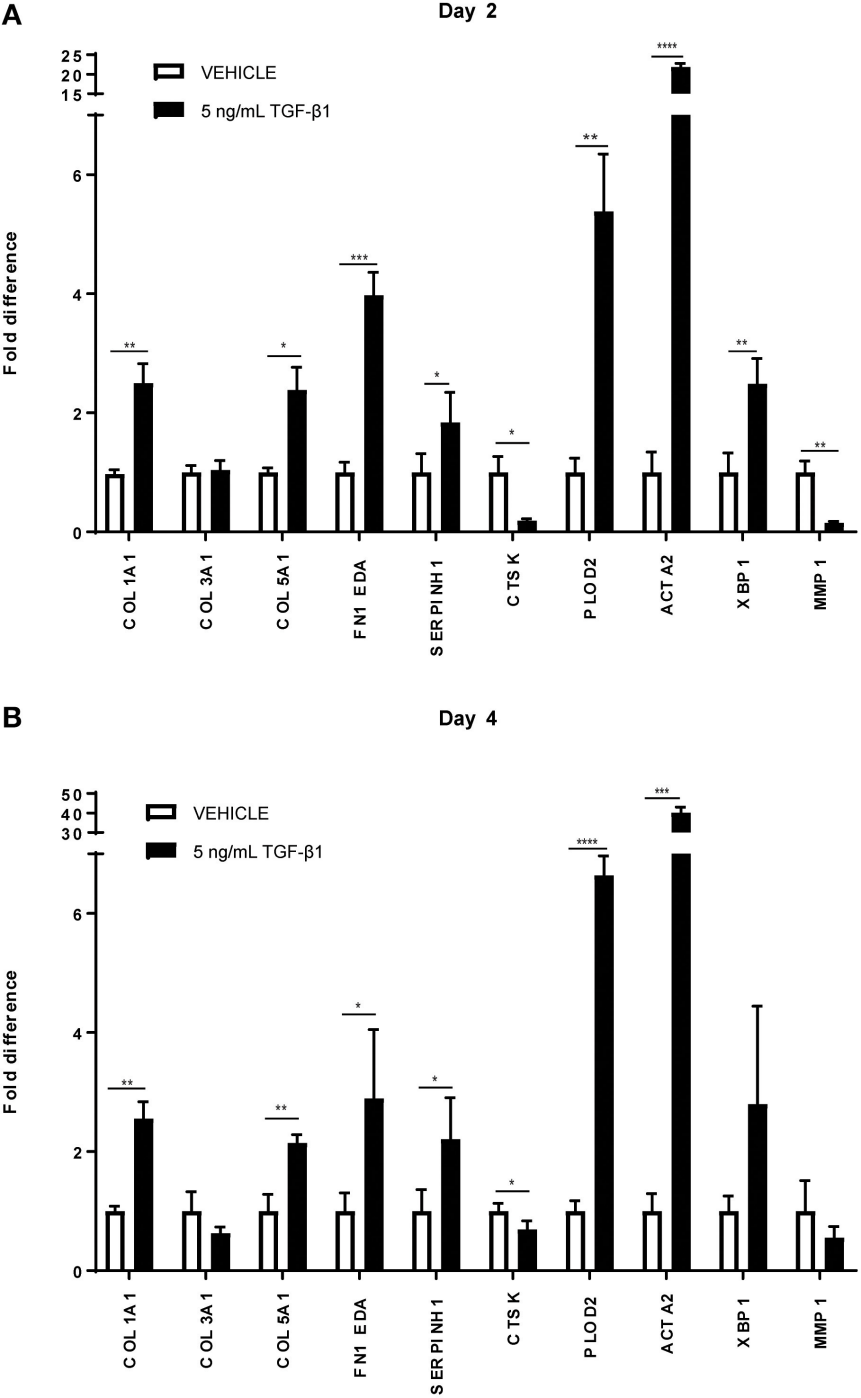
Relative mRNA expression of fibrosis markers in human dermal fibroblasts (*n* = 4) under TGFβ1 stimulation. mRNA expression of *COL1A1, COL3A1, COL5A1, FN1 EDA, SERPINH, PLOD2, ACTA2, XBP1, MMP1*, and *CTSK* in human dermal fibroblasts with 5 ng/mL TGFβ1 stimulation and without (=vehicle). **(A)** After 48 in culture. **(B)** After 96 h in culture. * = *P* ≤ 0.05; ** = *P* ≤ 0.01; *** = *P* ≤ 0.001; **** = *P* ≤ 0.0001.

It is well-known that TGFβ1 is a strong pro-fibrotic cytokine. To simulate fibrotic conditions, we added TGFβ1 to the primary adult human dermal fibroblasts, and checked whether the cells were responsive, both at day 2 and day 4. Indeed, as expected, a significant increase is seen in mRNA levels of genes encoding *COL1A1, COL5A1*, and *FN1EDA* (extracellular matrix proteins), *SERPINH1* and *PLOD2* (collagen-processing proteins), *ACTA2* (a marker for myofibroblasts) and *XBP1* (involved in the endoplasmic reticulum stress response), whereas a significant decrease was seen in mRNA levels of genes encoding for *MMP1* and *CTSK* (enzymes that are able to degrade collagen). We thus confirmed the fibrotic state of the cells (Figures 1A,B).

**Figure 2:**
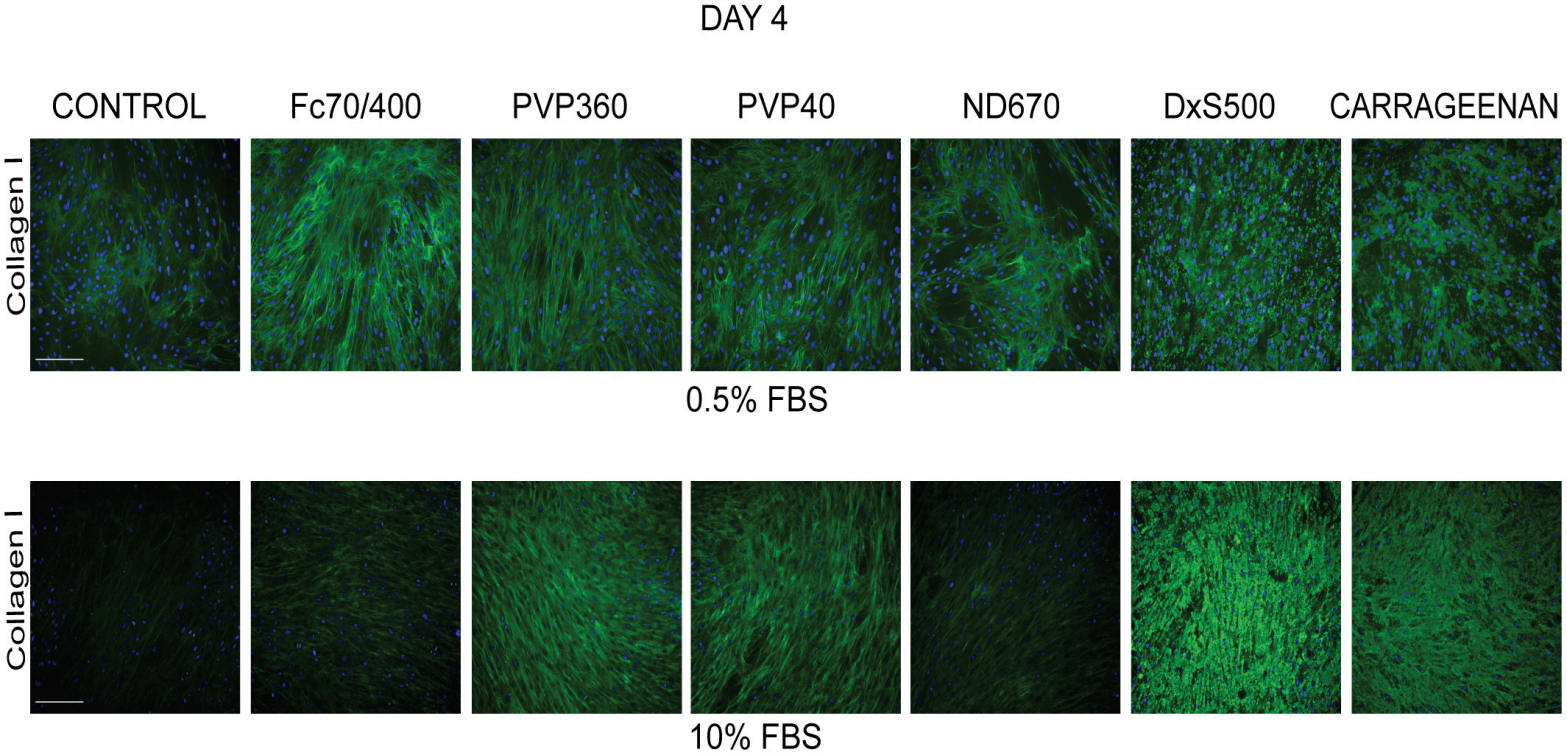
Effect of serum concentration on collagen I deposition. Immunofluorescence staining of collagen I in human dermal fibroblasts (*n* = 5) after 96 h in the presence of TGFβ1 in DMEM containing 0.5 and 10% of FBS and macromolecular crowders Fc70/400 mixture, PVP360, PVP40, ND670, DxS500, and CR. Showing 1 FVO; DAPI, Collagen I, magnification 10x, scale bars: 200 μm.

To test the validity of the Scar-in-a-Jar approach, we next investigated the effect of six macromolecular crowders (Fc70/400, PVP40, PVP360, ND670, DxS500, and CR) on collagen type I deposition under the same profibrotic conditions at day 4. We first tested the effect of the presence of 0.5 and 10% FBS in the culture medium, as there are contradictory data in the literature with respect to the effect of serum concentration on collagen deposition [34-36 + 39 *vs*. 38]. At 0.5% FBS, collagen deposition was seen at day 4 in all conditions (Figure 2). With the exception of ND670, all MMCs showed an increased collagen deposition in comparison with the control (=no added crowder). A different situation was observed with 10% FBS (Figure 2). Under this serum concentration, hardly any collagen deposition was seen in the control and with Fc70/400 and ND670. The crowders PVP40 and PVP360 showed a staining comparable with 0.5% FBS, whereas DxS500 and CR appeared to demonstrate increased staining in comparison with 0.5% FBS.

**Figure 3:**
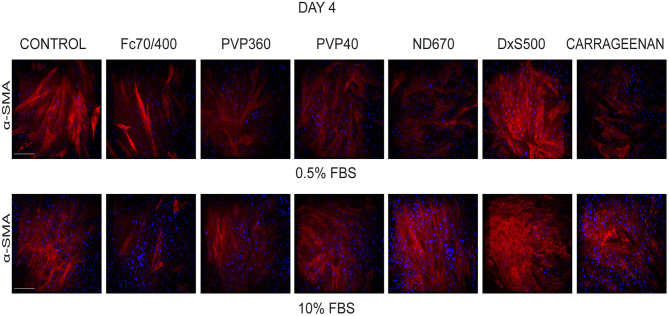
Effect of serum concentration on myofibroblast formation. Immunofluorescence staining of α-smooth muscle actin in human dermal fibroblasts (*n* = 2) after 96 h in the presence of TGFβ1 in DMEM containing 0.5 and 10% of FBS and macromolecular crowders Fc70/400 mixture, PVP360, PVP40, ND670, DxS500, and CR. Showing 1 FVO; DAPI, α-SMA; magnification 10x, scale bars 200 μm.

We also studied the transformation of fibroblasts into myofibroblasts at day 4 in the presence of 0.5 and 10% FBS under profibrotic conditions. In general, a more intense staining of α-SMA seemed to be seen with 0.5% FBS, indicating that myofibroblast formation proceeded more slowly in 10% FBS (Figure 3). Based on collagen deposition and α-SMA data we therefore decided to carry out subsequent experiments in 0.5% FBS.

**Figure 4:**
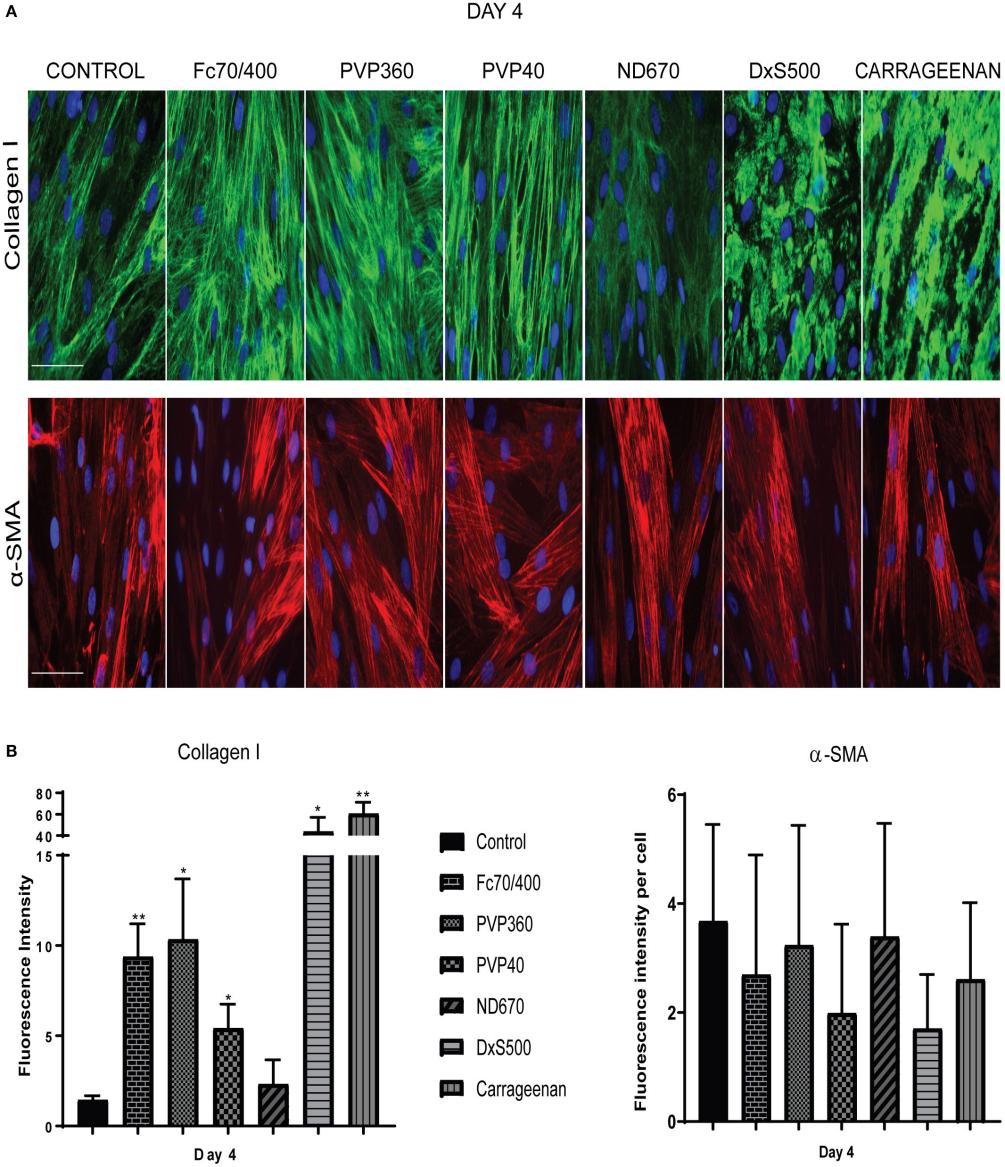
Patterns of collagen I deposition and α-smooth muscle actin cytoskeleton at high magnification as well as its quantification. **(A)** Immunofluorescence staining of collagen I and α-smooth muscle actin in human dermal fibroblasts (*n* = 3) after 96 h in the presence of TGFβ1, cultured with macromolecular crowders Fc70/400 mixture, PVP360, PVP40, ND670, DxS500, and CR. Showing 1 FVO; DAPI, collagen I, α-SMA; magnification 40x, scale bars 100 μm **(B)** Quantification of collagen I deposition (*n* = 5) and α- smooth muscle actin (*n* = 3) vs. control. * = *P* ≤ 0.05; ** = *P* ≤ 0.01.

Not only the amount of collagen that is deposited matters, but also the pattern of deposition. Increased magnification revealed, that with Fc70/400, PVP40, PVP360, and ND670 a reticular collagen network was formed, comparable with that of the control (Figure 4). In contrast, DxS500 and CR showed a highly granulated deposition. In addition, the myofibroblasts cultured under DxS500 and CR showed a less prominent α-SMA cytoskeleton (thinner stress fibers; Figure 4). Both observations point to the direction that DxS500 and CR interferes with normal fibrotic processes, and thus seems to be less favorable as a MMC in the Scar-in-a-Jar approach for testing anti-fibrotic drugs.

**Figure 5:**
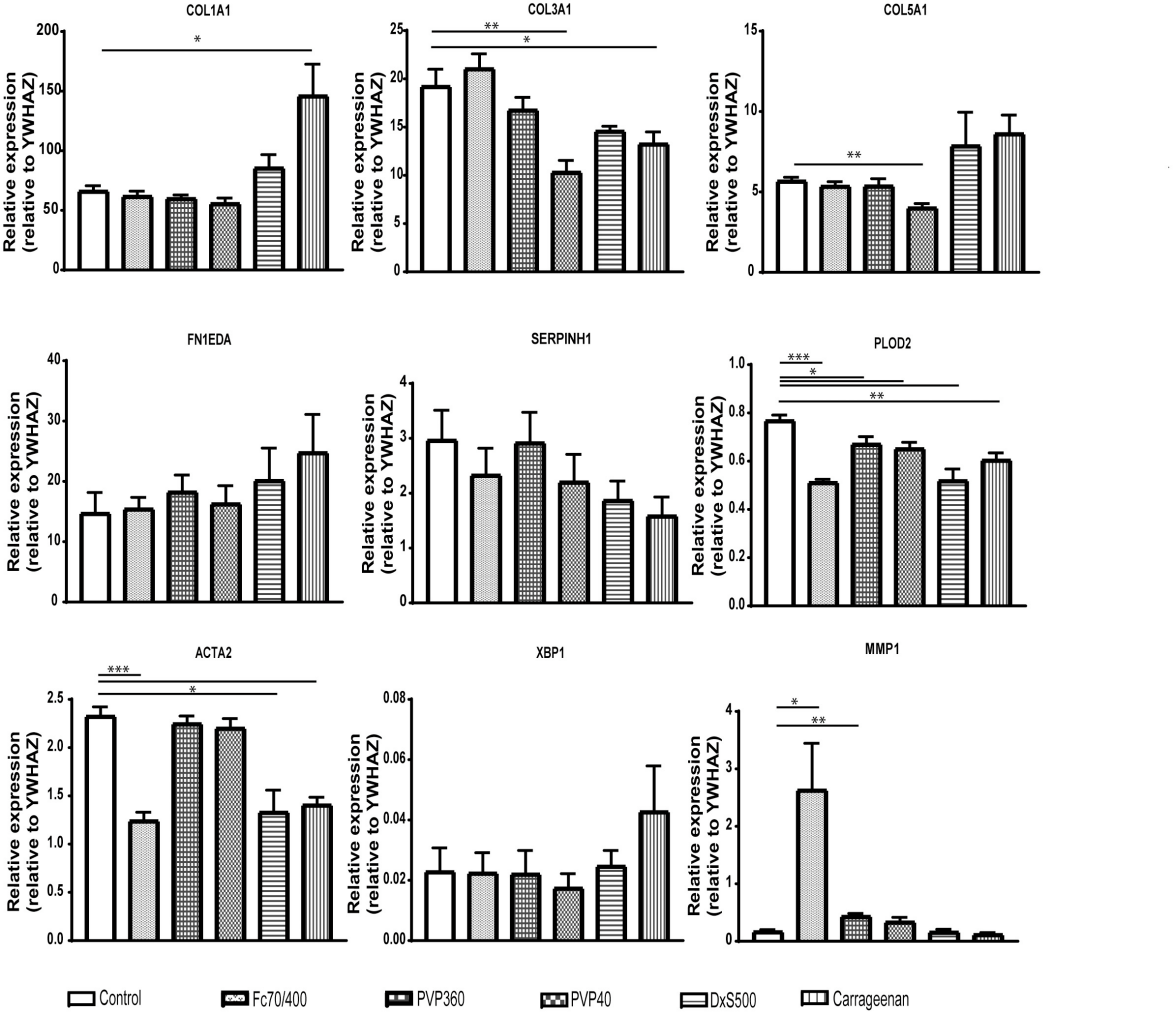
Effects of macromolecular crowders on fibrotic markers in dermal myofibroblasts. mRNA expression of *COL1A1, COL3A1, COL5A1, FN1EDA, SERPINH1, PLOD2, ACTA2, XBP1*, and *MMP1* in human dermal myofibroblasts (*n* = 4) after 96 h in the presence of TGFβ1, cultured with macromolecular crowders Fc70/400 mixture, PVP360, PVP40, DxS500, and CR vs. control. * = *P* ≤ 0.05; ** = *P* ≤ 0.01; *** = *P* ≤ 0.001.

To strengthen this conclusion, we also measured the gene expression of *COL1A1, COL3A1, COL5A1, FN1EDA, SERPINH1, PLOD2, ACTA2, XBP1*, and *MMP1* (Figure 5). mRNA levels of *ACTA2* and *PLOD2* were significantly decreased in the cells cultured with DxS500 and CR, indicating important phenotypical changes of the cells. The same was the case with Fc70/400, whereas a significant change was observed with PVP40 and PVP360 of *PLOD2* only. An increase of *COL1A1* expression was seen with CR, whereas the other MMCs did not affect *COL1A1* expression. A significant decrease in mRNA levels of *COL3A1* and *COL5A1* is seen in the presence of PVP40 and CR, whereas a significant increase of MMP1 is observed with Fc70/400 and PVP360. No significant differences in *XBP1* levels were observed between the MMCs and the control, indicating a comparable endoplasmic reticulum stress response. The mRNA data reveal that DxS500 and CR indeed have an effect on the fibrotic properties of the cells, and also discloses that Fc70/400 is a less favorable MMC in the Scar-in-a-Jar approach.

**Figure 6:**
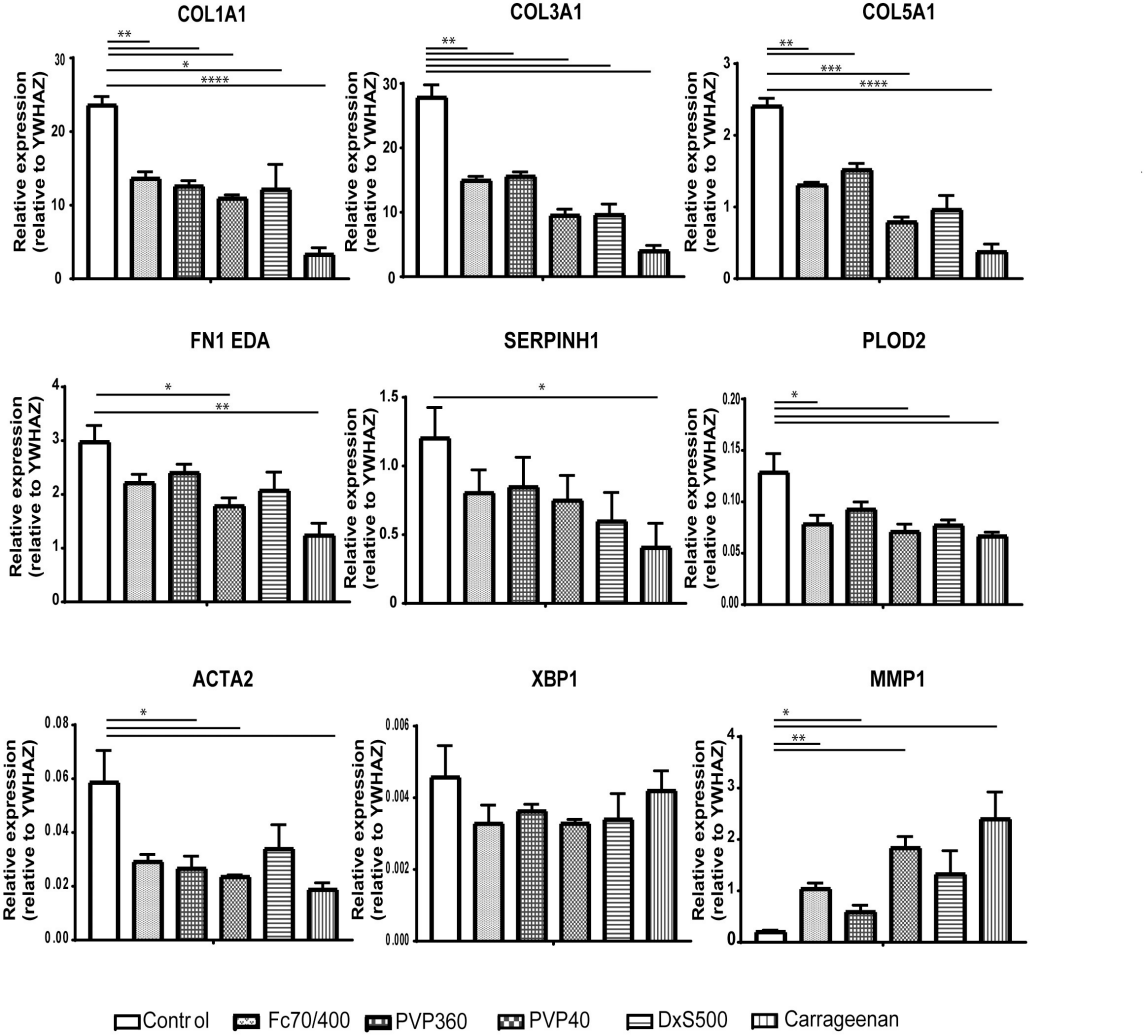
Relative mRNA expression of fibrosis markers in dermal fibroblasts (*n* = 4) after 48 h of culture. mRNA expression of *COL1A1, COL3A1, COL5A1, FN1EDA, SERPINH1, PLOD2, ACTA2, XBP1*, and *MMP1* in human dermal fibroblasts after 48 h without stimulation with TGFβ1, cultured with macromolecular crowders Fc70/400 mixture, PVP360, PVP40, DxS500, and CR vs. control. * = *P* ≤ 0.05; ** = *P* ≤ 0.01; *** = *P* ≤ 0.001; **** = *P* ≤ 0.0001.

**Figure 7:**
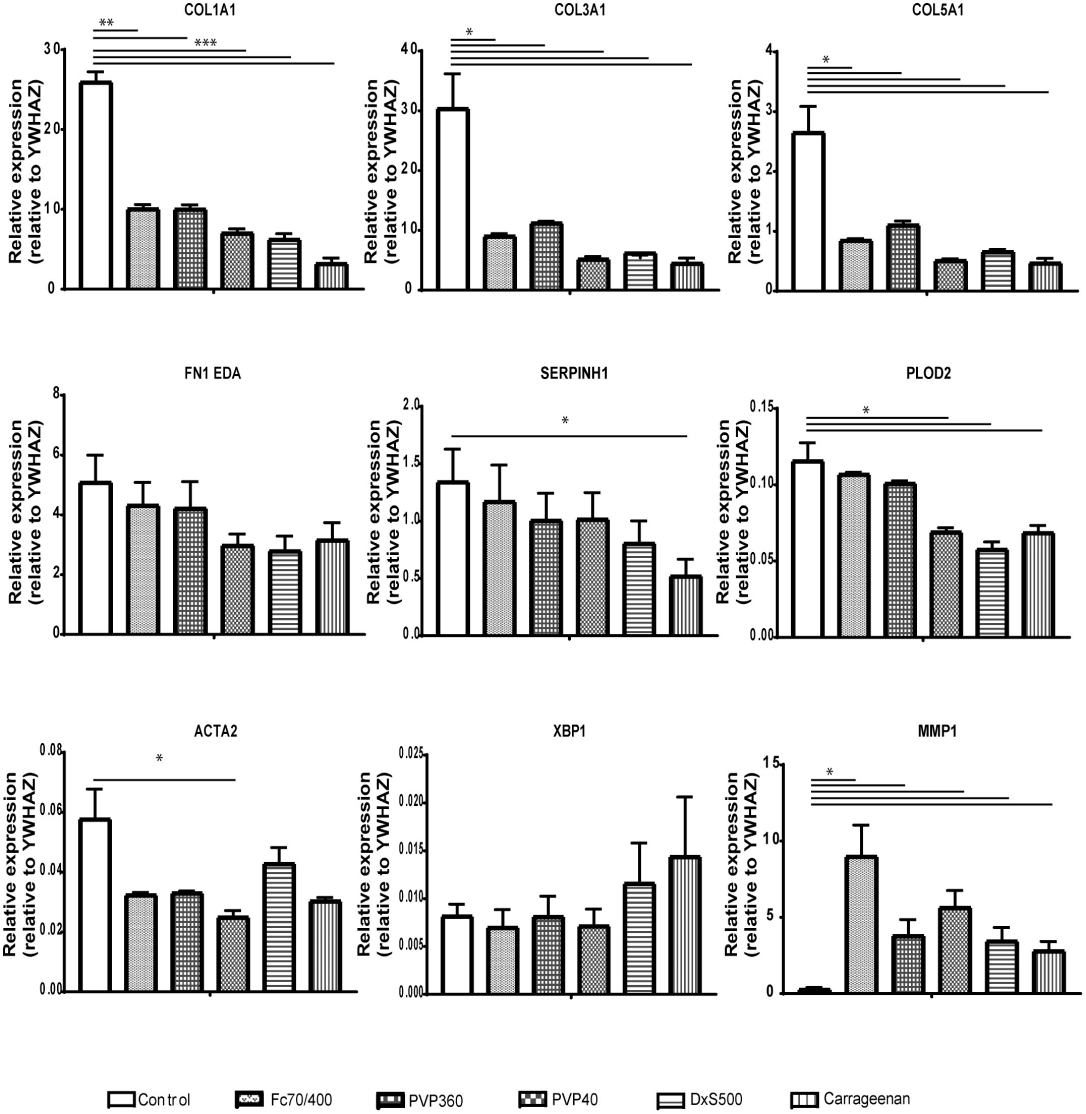
Relative mRNA expression of fibrosis markers in dermal fibroblasts (*n* = 4) after 96 h of culture. mRNA expression of *COL1A1, COL3A1, COL5A1, FN1EDA, SERPINH1, PLOD2, ACTA2, XBP1*, and *MMP1* in human dermal fibroblasts after 96 h without stimulation with TGFβ1, cultured with macromolecular crowders Fc70/400 mixture, PVP360, PVP40, DxS500, and CR vs. control. * = *P* ≤ 0.05; ** = *P* ≤ 0.01; *** = *P* ≤ 0.001.

Since some of the MMCs affect the phenotypical properties of fibroblasts under fibrotic conditions, we wondered whether MMCs have an effect on the phenotypical properties of non-stimulated fibroblasts. Interestingly, ***all***MMCs significantly decreased mRNA levels of *COL1A1, COL3A1, COL5A1*, and significantly increased mRNA levels of *MMP1*, both at day 2 and day 4 (Figures 6, 7). This underlines the importance of the addition of TGFβ1 into the medium to mimic pro-fibrotic conditions for the screening of anti-fibrotic drugs in the presence of MMCs.

Since under pro-fibrotic conditions ND670 does not result in an increased collagen deposition, and DXS500 and CR results in an aberrant collagen deposition and important phenotypical changes, and also Fc70/400 decreases mRNA levels of *ACTA2* and *PLOD2*, it seems that PVP40 and PVP360 are the best choice for a MMC to screen anti-fibrotic drug properties. As the preparation of culture medium with PVP360 is somewhat troublesome because of an increase in viscosity, we used PVP40 in the subsequent experiment.

**Figure 8:**
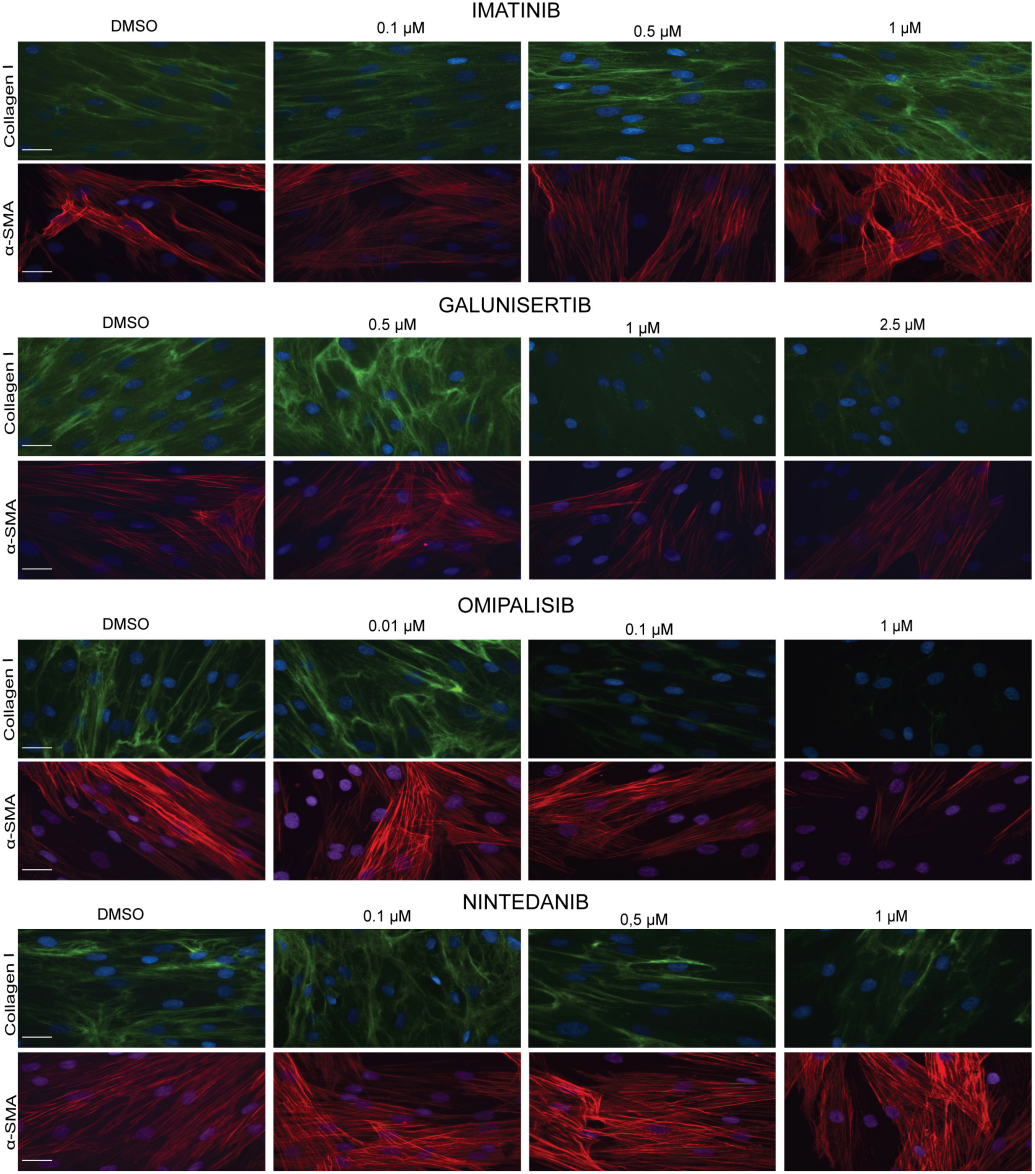
Effect of antifibrotic compounds in dermal myofibroblasts (*n* = 2). Immunofluorescence staining of collagen I and α- smooth muscle actin in human dermal fibroblasts after 96 h in the presence of TGFβ1 cultured with macromolecular crowder PVP40 and treated with anti-fibrotic compounds Imitanib, Galunisertib, Omipalisib, and Nintedanib. Showing 1 FVO; DAPI, collagen I, α-SMA; magnification 40x; scale bars 200 μm.

**Figure 9:**
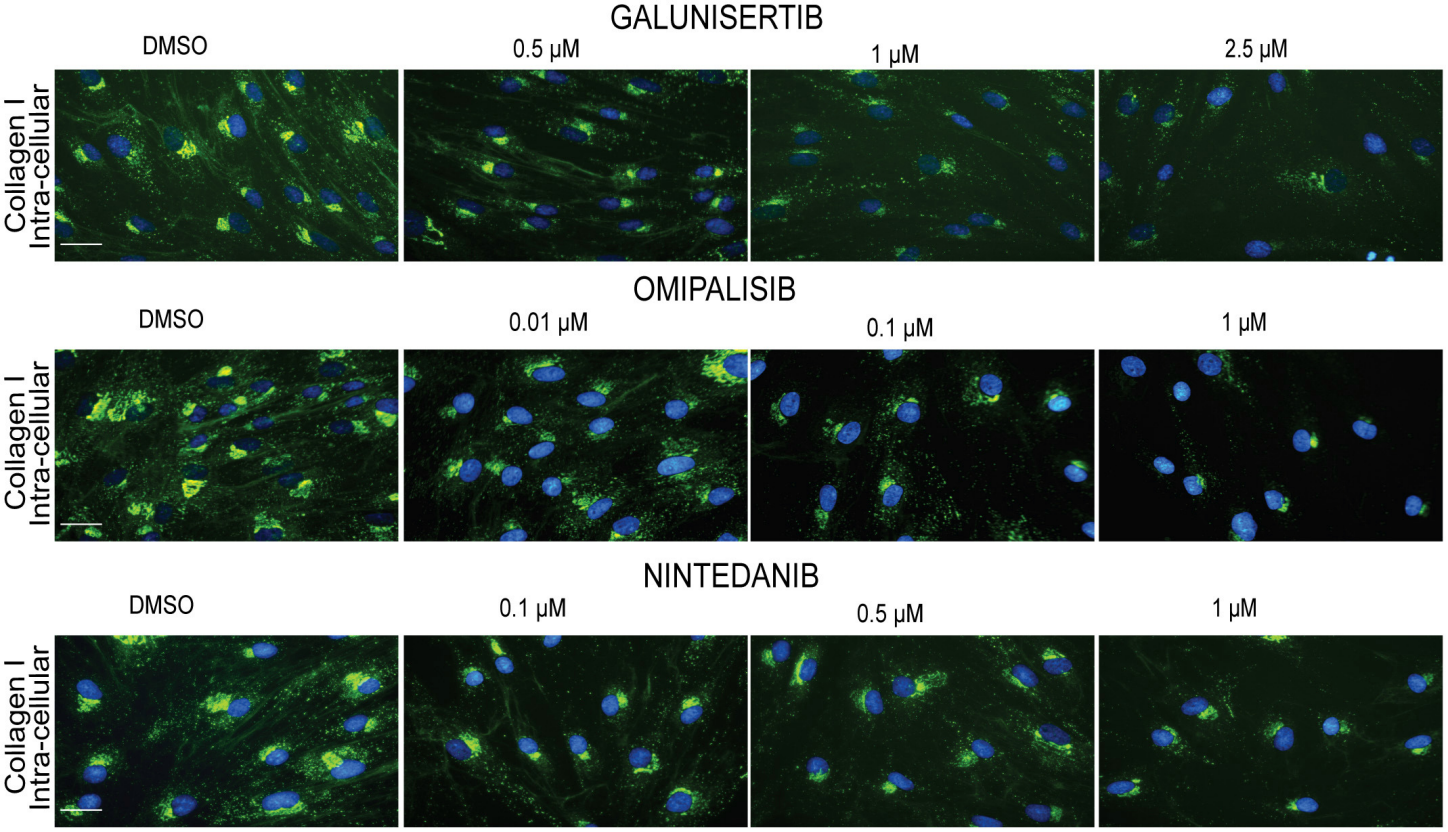
Effect of anti-fibrotic compounds on collagen I synthesis in dermal myofibroblasts. Immunofluorescence staining of intra-extracellular collagen I in human dermal fibroblasts after 48 h in the presence of TGFβ1 cultured with macromolecular crowder PVP40 and treated with anti-fibrotic compounds Galunisertib, Omipalisib and Nintedanib. Showing 1 FVO; DAPI, collagen 1, magnification 40x, scale bars 200 μm.

We visualized (Figure 8) collagen type I deposition and myofibroblast formation (positive staining for α-SMA) in the presence of various concentrations of Imatinib, Galunisertib, Omipalisib or Nintedanib with primary adult human dermal fibroblasts cultured for 4 days in DMEM containing 0.5% FBS, 50 U/L pen/strep, 0.17 mM ascorbic acid, 5 ng/ml TGFβ1 and 21.5 mg/ml PVP40. No decrease in collagen deposition or myofibroblast formation was seen with Imatinib, whereas a dose-dependent decrease in collagen deposition and myofibroblast formation is seen with Galunisertib and Omipalisib. On the other hand, Nintedanib showed a dose-dependent decrease of collagen deposition, but myofibroblast formation was unaffected. To verify whether the decrease in collagen deposition is due to a decreased synthesis of collagen, we fixed the cells with 0.5% Triton X-100. With this method mainly intracellular collagen can be visualized. Intracellular staining for collagen type I showed a marked decrease of collagen synthesis in the endoplasmic reticulum for Galunisertib, Omipalisib, and Nintedanib (Figure 9).

## Discussion

The addition of macromolecular crowders to cell cultures greatly facilitates the deposition of extracellular matrix molecules, in particular collagens. It enhances the cleavage of the collagen propeptides, which is normally a limiting factor in collagen fibrillogenesis, resulting in a shorter culture time at which collagen deposition can be seen by optical analysis. It has been used in tissue engineering to enhance tissue production or to enhance the performance of differentiated cells and/or stem cells. It also provides the opportunity to screen in an easy way drugs for their ability to attenuate collagen deposition. This is especially of interest in the field of fibrosis, a pathology that is characterized by an excessive deposition of collagen type I; it is known as the Scar-in-a-Jar approach. When implementing this approach into our laboratory for drug screening, we observed a major hiatus in the validation of this system in the literature.

The majority of macromolecular crowding studies have been carried out with fibroblasts under non-fibrotic conditions. However, the deposition of collagen in fibrosis is mainly done by myofibroblasts, i.e., fibroblasts that are activated by pro-fibrotic cytokines such as TGFβ1. But studies that took this into consideration used embryonic fibroblasts, although it is known that wounded embryos heal without scarring. Furthermore, it has not been tested whether cells become phenotypically different in the presence of MMCs, as the focus was on extracellular matrix deposition. For our validation studies we therefore used adult human dermal primary fibroblasts cultured in the presence of TGFβ1, tested the effect of serum concentration in the medium on collagen deposition and myofibroblast formation, tested the effect of the different MMCs on the phenotypical properties of stimulated and unstimulated fibroblasts, and finally tested the system with the most favorable MMC with four anti-fibrotic drugs.

We first tested the effect of 0.5 and 10% FBS on collagen deposition and myofibroblast formation in the presence of TGFβ1. We found significant collagen deposition with 0.5% FBS with all six MMCs, whereas with 10% FBS hardly any collagen deposition was found with Fc70/400 and ND670. Furthermore, we observed a delayed myofibroblast formation in 10% FBS. It has been reported that the presence of 0.5 or 10% bovine serum has no significant effect on the amount of deposited collagen in the presence of DxS500, Fc70/400 or CR when cultured for 2–4 days under 20% oxygen tension under non-fibrotic conditions (34–36, 39), whereas another report found increased levels of collagen at 0.5% FBS with CR (38). The latter was explained by an enhanced degradation of collagen due to the presence of matrix metalloproteinases in the used serum (only CR was tested) (38). We found that collagen deposition in 10% serum is a MMC-specific phenomenon (present with PVP40, PVP360, DxS500, CR, absent with Fc70/400 and ND670); therefore, the suggested presence of matrix metalloproteinases cannot be held responsible for the presence or absence of collagen. Myofibroblast formation was not studied previously. Our data on collagen deposition and myofibroblast formation show that 0.5% FBS has a superior performance compared to 10% FBS. However, the collagen deposited by DxS500 and CR showed an aberrant pattern, whereas a normal pattern was seen with Fc40/700, PVP40, PVP360, and ND670, but the latter did not show an enhanced deposition compared to the control.

Drug screening provides the most reliable data when carried out in an environment in which the cells exhibit their normal phenotype. We found that all MMCs (we excluded ND670 because of the low collagen deposition) have serious effects on the phenotypic properties of non-stimulated fibroblasts, whereas this is much less obvious (but still present in some MMCs) when the cells are cultured under pro-fibrotic conditions. CR, DxS500, and Fc40/700 affected several major phenotypical properties of the cells when cultured under pro-fibrotic conditions, whereas PVP40 and PVP360 showed fewer perturbations. Changes in phenotypical properties by MMCs (DxS500, Fc40/700) have previously been reported for corneal fibroblasts and mesenchymal stem cells derived from bone marrow and fat tissue (10, 11, 34), but it was so far not studied in pro-fibrotic conditions. Clearly, PVP40 or PVP360 should be used as MMC in the context of drug screening.

Trichostatin, Ciclopiroxolamine and PcP56 have been tested previously as drugs with embryonic lung fibroblasts under pro-fibrotic conditions in the Scar-in-a-Jar system (6, 31), and hepatocyte growth factor and Botox type A with adult immortalized focal fold fibroblasts (32). We have here tested Imatinib, Galunisertib, Omipalisib and Nintedanib, with PVP40 as the molecular crowder and primary adult human dermal fibroblasts as cell type. Imatinib inhibits c-abl kinase and blocks the PDGF receptor (51). Although it attenuates fibrosis in various animal models, we did not see an effect on collagen deposition by dermal fibroblasts. This is in line with previous data obtained with cardiac fibroblasts (52). Galunisertib, by being a selective inhibitor of TGFβR1, inhibits phosphorylation and activation of SMAD2/3 (53). Although it is under investigation for the treatment of different cancers, it was shown recently that it has also antifibrotic properties (47, 54). Indeed, collagen deposition was not observed in our test system. The latter was also observed with Omipalisib (GSK2126458), a potent inhibitor of phosphatidylinositol 3-kinases (PI3Ks) and mechanistic target of rapamycin (mTOR) which was also developed in the oncology setting, and is recently being tested in fibrosis (55). Both Galunisertib and Omipalisib also inhibited myofibroblast formation. Finally, Nintedanib showed reduced collagen deposits whereas myofibroblast formation was unaffected. Both observations are in line with published records (56, 57).

In conclusion, we found that the Scar-in-a-Jar approach should best be carried out under profibrotic conditions (the presence of TGFβ1) in a culture medium with 0.5% FBS and with PVP40 (or PVP360) as the macromolecular crowder. The data obtained with Imatinib, Galunisertib, Omipalisib, and Nintedanib are comparable with previously published data that were obtained without macromolecular crowder. It shows that the Scar-in-a-Jar approach as described in this paper is a reliable test system to screen antifibrotic drug properties.

## Data Availability Statement

The raw data supporting the conclusions of this article will be made available by the authors, without undue reservation.

## Author Contributions

NP, PO, and RB conceived the presented idea. NP, MB, and RB conceived the experimental setup. NP performed all experiments and analyzed all data. MB and EB contributed with discussion and data analysis tools. NP and RB drafted and wrote the manuscript. PO and RB revised and approved the manuscript. All authors contributed to the article and approved the submitted version.

## Conflict of Interest

The authors declare that the research was conducted in the absence of any commercial or financial relationships that could be construed as a potential conflict of interest.

## Abbreviations

*ACTA2*: alfa-smooth muscle actin
BSA: bovine serum albumin
*COL*: collagen
*CTSK*: cathepsin K
DMEM: Dulbecco's modified Eagle medium
DxS500: dextran sulfate 500
ECM: extracellular matrix
FBS: fetal bovine serum
Fc70: Ficoll PM 70
Fc400: Ficoll PM 400
*FN1EDA*: fibronectin 1 containing extra domain A
Fc70/400: a mixture of Ficoll 70 and 400
MMC: macromolecular crowding
MMCs: macromolecular crowders
*MMP1*: matrix metalloproteinase 1
ND670: neutral dextran 670
*PLOD2*: lysyl hydroxylase 2
PVP40: polyvinylpyrrolidone 40
PVP360: polyvinylpyrrolidone 360
*SERPINH1*: heat-shock protein 47
α-SMA: α-smooth muscle actin
TGFβ1: transforming growth factor β1
*XBP1*: X-box binding protein 1
*YWHAZ*, tyrosine 3-monooxygenase/tryptophan 5-monooxygenase activation protein: zeta isoform.
